# 4135 Digital Mental Health Interventions for PTSD & Resilience: A Systematic Review

**DOI:** 10.1017/cts.2020.195

**Published:** 2020-07-29

**Authors:** Marie Torres, Alfonso Martinez-Taboas, Karen G Martinez

**Affiliations:** 1 University of Puerto Rico-Medical Sciences Campus; 2 Carlos Albizu University

## Abstract

OBJECTIVES/GOALS: 1) In this literature review we want to explore the literature on DMHI (Interventions delivered via digital technologies, such as smartphones, websites, or text messaging), specifically designed to treat Posttraumatic Stress Disorder (PTSD), and/or to promote positive change & resilience after trauma. 2) We also want to evaluate the literature in terms of the theoretical model used in each DMHI, engagement, effectiveness, & potential harms/challenges. METHODS/STUDY POPULATION: We will review the literature that describes DMHI for PTSD, resilience, & positive change in persons exposed to psychological trauma (Exposure to actual or threatened death, serious injury, or sexual violence, as defined by the fifth edition of the Diagnostic & Statistical Manual of Mental Disorders). We will review the following databases: PsychINFO, EBSCOhost, PubMed, & PsychiatryOnline. The following inclusion criteria will be used: 1) Interventions delivered by computer, smartphones, or online, 2) studies published between 1999-2019. Exclusion criteria will include reviews, opinion, or discussion articles, & unpublished works. RESULTS/ANTICIPATED RESULTS: We expect to find that the most popular therapeutic model for DMHI is cognitive behavioral therapy. We also expect to find a higher number of web-based interventions, as opposed to phone-based interventions, or other types of DMHI. We also expect to find variable drop-out rates, low engagement, & small to moderate effect sizes. DISCUSSION/SIGNIFICANCE OF IMPACT: We expect our contribution to center on evaluating available DMHI for psychological trauma. This systematic literature review is expected to provide scientific justification for the development (or validation), & implementation of a DMHI that takes into account the results of previous studies. This contribution is expected to be significant because it will help in choosing, or developing an effective future intervention with DMHI. CONFLICT OF INTEREST DESCRIPTION: There is no conflict of interest in this study.

